# Influence of Integrated Membrane Treatment on the Phytotoxicity of Wastewater from the Coke Industry

**DOI:** 10.1007/s11270-018-3794-1

**Published:** 2018-04-30

**Authors:** Marzena Smol, Dariusz Włóka, Maria Włodarczyk-Makuła

**Affiliations:** 1 Mineral and Energy Economy Research Institute, Polish Academy of Sciences, 31-261 Cracow, Poland; 2 Institute of Environmental Engineering, Faculty of Infrastrcture and Environment, Czestochowa University of Technology, 42-200, Czestochowa, Poland; 3 Department of Chemistry, Water and Wastewater Technology, Faculty of Infrastrcture and Environment, Czestochowa University of Technology, 42-200, Czestochowa, Poland

**Keywords:** Coke wastewater, Wastewater treatment, Gemination inhibition, Phytotoxicity, Toxity test, Polycyclic aromatic hydrocarbons (PAHs)

## Abstract

In this paper, coke wastewater that had passed through biological and integrated membrane processes (filtration on sand bed—reverse osmosis) was chosen to assess the phytotoxicity of selected industrial wastewater with regard to the test plant—*Vicia faba*. An innovative research technique in vitro test was conducted in a large scale phytothrone chamber on two matrices: cotton and Murashige and Skoog Basal Medium (MSBM). The toxicity of wastewater was evaluated for samples: (1) treated in the treatment plant by biological processes, (2) filtrated through a sand bed and filtrated (3) reverse osmosis (RO) membrane. The results showed that there is a noticeable correlation between increasing concentrations of wastewater and seed germination of the test plant. Although the wastewater collected from the coke plant was treated biologically, it showed very high levels of germination inhibition (90–98% for cotton matrix and 92–100% for MSBM matrix) and strong toxic effects. The wastewater collected from the coke plant showed a significantly greater phytotoxic effect compared with those obtained from the effluent treated on a sand bed and in RO. However, wastewater, even after treatment on a sand bed (reduction of COD—39%, TN—46%, TOC—42%, TC—47%, SS—50%, 16PAHs—53%), was still toxic and germination inhibition was in the range of 24–48% for the cotton matrix and 14–54% for the MSBM matrix. The toxicity of wastewater treated in the membrane process was the lowest (reduction of COD—85%, TN—95%, TOC—85%, TC—86%, SS—98%, 16PAHs—67%). The germination inhibition was in the range of 4–10% for the cotton matrix and 2–12% for the MSBM matrix. These samples are classified as non-toxic or slightly toxic to the model plant. The present study highlights the necessity of monitoring not only the basic physical and chemical indicators (including the level of toxic substances as PAHs), but also their effect on the test organisms in wastewater samples.

## Introduction

One of the most hazardous industrial effluents is wastewater generated during the process of coke production (Macherzyński et al. 2014) and the treatment and processing of coking by-products (Zhao et al. 2015). Coke wastewater contains sizable amounts of ammonium salts and compounds such as phenols, oils, tars, suspensions, polycyclic aromatic hydrocarbons (PAHs), toxic organic nitrogen compounds, cyanide, ammonia and hydrogen sulphide (Pillai and Gupta 2016). Raw coke wastewater contains the above toxic impurities and it cannot be sent to the receiver without purification (Włodarczyk-Makuła et al. 2016). Coke wastewater is initially directed to treatment installations located in the area of the coking plant. The most widely used method in such installations is a biological process (Wu et al. 2016), which in many cases is insufficient. It is recommended to use of integrated systems connecting classic unit processes technology used in the treatment (biological, chemical and physical) (Bodzek and Dudziak 2006). The membrane techniques are high effective in the removal of pollutants from aquatic solutions (Kamińska et al. 2016). However, even after integrated treatment, coke wastewater can be still toxic for evrironment. One of the ways to assess the degree of toxicity of wastewater, apart from the determination of the physical and chemical indicators (Paździor et al. 2016; Generowicz et al. 2011), is the toxicity test (da Costa Filho et al. 2016). Currently, tests based on plant bioindicators (Rorat et al. 2014) are often recommended as an effective and affordable method of assessing the toxicity (Placek et al. 2016) and genotoxicity of environmental samples with a sensitivity similar to tests using mammalian cells (Obidoska et al. 2015). In this paper, coke wastewater after biological treatment in coke plant was directed to integrated membrane processes (filtration on a sand bed—reverse osmosis), and the phytotoxicity of wastewater on a test plant *Vicia faba* was assessted.

## Characteristics of Wastewater Generated in Coke Plants

Coke production is based on high-temperature pyrolysis of coal in batteries of coke ovens (Ghose 2002). As a result of the degassing process, coke (constituting a 70–80% proportion of all the coking products) and the raw coke oven gas are obtained. The by-products are tar, coking benzol (depending on the technology used), products of the desulphurisation of and binding of ammonia from the coke oven gas through such products as as ammonium sulphate (Martínková and Chmátal 2016).

Coke plants are facilities which consume large amounts of water and generate highly polluted wastewater from the production proces (Dong and Zhang 2010). The wastewater generated in the process of coke production and purification, and the processing of coking by-products can be divided into the following types:

- coal water (ammonia) resulting from the condensation of derived water vapour (from coke oven gas, during the cooling operation);
- outflows from the treatment by-products of degassing;
- effluent from wet coke quenching;
- liquids from the rectification and condensing of benzene;
- leachate resulting from the processing of tar and hydrogen sulphide;
- condensates of water vapour consumed for the direct heating of media in technological processes (e.g. stripping ammonia from the coal water and benzene from the wash oil);
- outflows from the closures of hydraulic gas pipes;
- condensates from the cleaning of implanted impurities from the equipment and ducts by means of water vapour;
- outflows from the periodic cleaning of floors, equipment, devices, etc. (Alexandersson 2007; BAT 2005).

Coke wastewater is a complex industrial wastewater present in most steelworks (Vázquez et al. 2007). The main factor determining the amount of wastewater generated during the process of coking coal is the amount of processed raw material. The amount of wastewater is also dependent on the type of coal used in the process of gas purification, the technologies for the recovery of by-products and the water/wastewater management model used (Qi et al. 2007). The average quantity of process wastewater arising from the coke plant ranges from 0.15 to 0.35 m^3^/Mg of coal. From this, one can calculate that between 0.35 and 0.45 m^3^ of wastewater arises per tonne of coke produced (Bartkiewicz 2008). The basic pollutants in coke wastewater include toxic compounds such as ammonia salts (CN^−^, SCN^−^), phenols, oils, tars, suspensions, polycyclic aromatic hydrocarbons (PAHs) (Włóka et al. 2017), toxic organic nitrogen compounds, cyanide (Oulego et al. 2014) ammonia and hydrogen sulphide (Smol and Włodarczyk-Makuła 2015). It is worth noting that coke wastewater is classified as an onerous industrial effluent. Contaminants present in the post-process coke wastewater are a source of indirect emissions of PAHs in the case of coke wastewater used to supplement the circulation of wet quenching of coke. The composition of coke wastewater from different plants is shown in Table 1. Individual concentrations of the components listed vary according to the type of coal used and the modifications made to each specific process.

**Table 1:** Composition of coke wastewater

Parameter	Unit	Lai et al. 2007	Vázquez et al. 2007	Zhao et al. 2009a, b	Zhao et al. 2010	Wei et al. 2012	Mielczarek et al. (2014a, b)	Madeła and Dębowski 2006	Smol et al. 2014b
Temperature	°C	30	–	28–38	–	–	–	36	47
pH	–	7.2	8.1	6.53–8.04	–	–	9.1–9.4	7.5–9.1	7.56
COD	mg/L	750	1100	1182–3310	2236	1676	3489–4520	–	6080.4
BOD_5_	mg/L	45	579	–	–	–	50	–	–
TOC	mg/L	195	–	–	–	–	–	–	386.1
TC	mg/L	100	–	–	–	–	–	–	496.9
Conductivity	μS·cm^−1^	2200	7100	–	–	–	1700–8410	–	–
Phenols	mg/L	–	207	331–1078	300	249	381–534	260–3000	–
NH_3_-N	mg/L	–	–	49–488	–	106	–	–	–
NH_4_^+^-N	mg/L	–	688	265.9	211	–	132–491	980–6500	168.0
NO_2_-N	mg/L	–	–	–	–	–	–	–	
Total nitrogen	mg/L	–	–	110–617	370	–	1820	–	609.1
Turbidity	NTU	–	–	1.8–528	–	–	–	–	375.1
Alkalinity	mg/L	–	250	224–916	–	–	–	–	–
Colour	–	–	–	1250–1900	–	–	–	–	–
Cyanide	mg/L	–	–	–	24	9.65	11–27	10–100	–
Oil and tar	mg/L	–	–	–	–	–	–	100–240	–

According to the legal restrictions, coking enterprises are obliged to carry out coke production with the use of Best Available Techniques (BAT). In Directive 96/62/EC on integrated pollution prevention, called the IPPC (Integrated Pollution Prevention and Control), standard BAT determines limits to emission levels for large industrial plants, including the coking plant. The characteristics of BAT in the European coke industry were concluded in a BREF document (Best Available Techniques Reference Document on the Production of Iron and Steel), Seville 2000. In accordance with BAT standards, it is essential that those available technological and technical solutions should be used which contribute to minimising the emission of pollutants into the air and soil-water environment. According to Polish law, the coke companies are required to obtain an integrated permit (Smółka et al. 2003) as the installation can be a source of significant pollution of the natural environment (Włóka et al. 2013).

Water and wastewater management in coke plants depends on the technical and technological solutions used. Wastewater discharged from large installations is subjected to a multi-step purification process, and wastewater from smaller installations, after initial pre-treatment, is returned to the technological cycle at the plant (for wet quenching of coke) or discharged into municipal sewage treatment plants. Raw coke wastewater contains the toxic compounds indicated above which are very harmful to human health, and it cannot be introduced into receivers (natural watercourses and sewers) without treatment. In most cases, wastewater is initially treated in biological treatment installations located in the area of the coking plant. However, after the biological processes, the quality of coke wastewater is often unsatisfactory (Smol et al. 2014b). Therefore, it is necessary to develop sustainable concepts for treating wastewater (Dudziak and Gryta 2013) properly in order to avoid any adverse technological, environmental and ecological impacts on the receiver (Smol et al. 2016a, b).

## Materials and Methods

### Experimental procedure

**Fig. 1:**
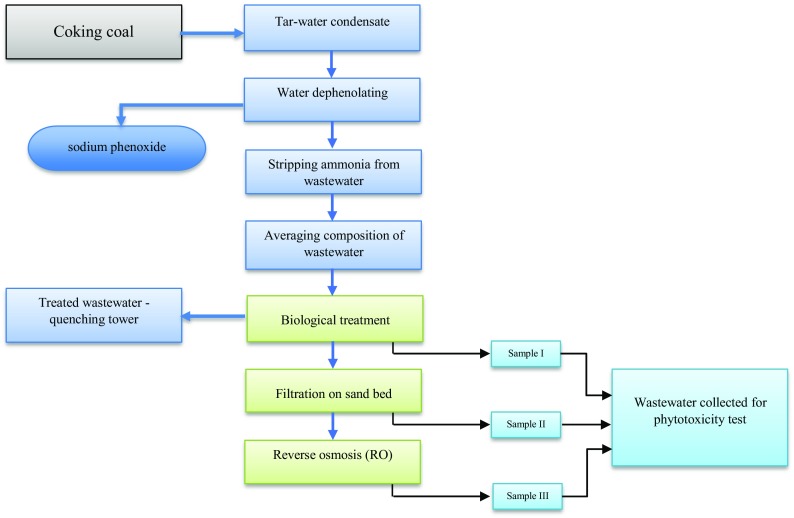
Stages of coke wastewater treatment

Coke wastewater samples were collected from a plant located in Silesia, southern Poland. The production capacity of the plant is 600,000 t of coke per annum. Wastewater was treated in the treatment plant by biological processes involving the separate denitrification, nitrification and oxidation of organic carbon. A diagram showing the individual stages of coke wastewater treatment in the study plant and the location of sampling is shown in Fig. 1.

Coke wastewater after the biological treatment in the coke plant was directed to laboratory experiment. According to high effectivnes in the removal of pollutants from aquatic solutions, adsorption (Dudziak and Werle 2016) and membrane processes (Tomaszewska and Bodzek 2013a, b) were used in this study. Wastewater samples were taken from the tank and stored in 10 l containers at a temperature of 10 °C. Coke wastewater was filtered on a sand bed (pre-filtration) in the laboratory. The sand bed was a cylinder shaped container filled with three layers of gravel and sand. The layers were composed of bottom layer gravel *Ø* = 1.0 cm, middle layer gravel *Ø* = 0.6 cm and upper layer sand *Ø* = 0.1–2.0 mm. The total depth of the filter layers was 50 cm, and the volume of the filter bed was 25 L. After pre-filtration, wastewater was directed to the membrane module for the main filtration—a high-pressure cross-flow GE-Water SEPA CF Membrane Cell (Bohdziewicz et al. 2016) with one nylon RO membrane (ADF). The setting operated in cross-flow closed system mode in which the retentate was recycled to the feed tank (Smol et al. 2016a, b). The pH range of the membrane operation is 1–11 with a maximum temperature of 50 °C and salt rejection for NaCl of 95.5%. The filtration area was 144 cm^2^, the transmembrane pressure of the process remained at the value of 2.0 MPa, the linear flow velocity over the membrane surface was 2 m·s^−1^, and the permeate flux was equal to 4.59·10^−6^ m^3^/m^2^·s.

The concentration of selected indicators in the wastewater was measured, comprising the concentration of 16 polycyclic aromatic hydrocarbons listed by the U.S. Environmental Protection Agency, pH, temperature, total nitrogen (TN), chemical oxygen demand (COD), total organic carbon (TOC), total carbon (TC) and suspended soils (SS). Tests of indications were performed in four replicates. Coke wastewater was analysed in accordance with generally accepted methodologies (Dojlido et al. 1999) briefly described in Smol et al. (2017).

The standard limit values of the selected indicators defined in Polish regulations are shown in Table 2.

**Table 2:** The limit values of selected indicators in wastewater (Smol et al. 2014a)

Indicator	Unit	Indexes of sewage pollution which is to adischarged to a natural receiver*	Indexes of sewage pollution which is directed to sewers**
pH	–	6.5–9.0	6.5–9.5
temperature	**°**C	35	35
Ammonium nitrogen	mg N-NH_4_^+^/L	10	100^1)^ 200^2)^
Nitrate nitrogen	mg NO_3_^−^/L	30	10
COD	mg O_2_/L	125	3)
TOC	mg C/L	30	3)
TC	mg C/L	ns.	ns.
SS	mg /L	35	3)
16PAHs	μg/L	ns.	200***

ns, not standardised

*Journal of law 2014 item.1800

**Journal of law 2006 no. 136, item. 964

***calculated on the basis of carbon content

^1)^For wastewater discharged to the treatment plant for an area with a population > 5000

^2)^For wastewater discharged to the treatment plant for an area with a population ≤ 5000

^3)^The values of indicators should be based on the permissible load of these pollutants for individual treatment plants

#### Wastewater treatment methods

The test was designed to determine the impact of coke wastewater on the germination of the test plant, broad bean (*Vicia faba*). The first wastewater sample was taken from the coke treatment plant: sample I—after the biological treatment. The second and third samples were prepared in the laboratory: sample II—after filtration through the sand bed, sample III—after reverse osmosis. All tests were conducted in three replicates.

#### Wastewater samples for germination tests

An innovative research technique, in vitro tests, was used for the study of phenomena resulting from the toxicity of pollution on plants. The toxicity test was set up in the large scale phytothrone chamber BioGenet. The prepared experiment was hold, with use of four experimental blocks. Each block includes two sets of Petri plates with two types of incubation matrix. Plates in first block was filed only with the incubation matrix (control sample). Wastewater was added to the remaining blocks, according to the instructions outlined in the previous paragraph (sample I; sample II; sample III).

#### Preparation of Seeds for the Test

Tests were performed on selected (without blemish, of similar size) seeds of the test plant—*Vicia faba*. All seeds came from one producer and from the same series. Initially, the seeds were stored at 4 °C for 24 h. Then, the seeds were sterilised in a mixture of ethyl alcohol 96% and hydrogen peroxide 34% (1:1) for 7 min, washed ten times in 200 ml of sterile distilled water and further placed by sterile tweezers (well heated above a flame) into sterile Petri dishes with a cotton matrix and sterile Petri dishes with a prepared matrix of Murashige and Skoog Basal Medium (MSBM). In each variant, 50 seeds were placed on the plate, (three repetitions). All operations were performed in a chamber with laminar air flow.

#### Germination Conditions

Seed germination was carried out for 72 h under controlled conditions in the phytothrone chamber, at a temperature of 21 °C (day) and 18 °C (night). Plants were grown under artificial illumination (fluorescent lamps) in an all day cycle. After the incubation period, both seeds that had germinated and those that had not germinated were counted.

#### Statistical Analysis

The membrane process capacity was enabled by the determination of volumetric permeate flux for deionised water—*J*_w_ and simulated solutions *J*_v_ calculated based on formula incicated in Bohdziewicz et al. 2014. The efficiency of the filtration was determined on the basis of the retention ratio [%]. For the membrane techniques, retention coefficient [*R*, %] is used (Smol and Włodarczyk-Makuła 2017).

Average values for the germinating seeds were calculated for each sample, based on a control sample. The toxicity indicator, inhibition of germination (*inhibition*), was determined according to the following formula:

$$ I=\frac{A-B}{A}\cdot inhibition=100\kern0.5em \left[\%\right] $$

where:

I: inhibition of germination (*inhibition*) [%],
A: seed germination in the control sample,
B: seed germination in a test sample (Trojanowska-Olichwer 2013).

In order to evaluate a statistical valid differences between samples, the one-way ANOVA test, followed by Tukey’s post hoc range analysis, was performed. This procedure was conducted on StratSoft STATISTICA software.

## Results and Disscusion

### The Removal Efficiency of Selected Pollution from Coke Wastewater

The composition of the raw and treated coke wastewater is shown in Table 3.

**Table 3:** Changes in the physical and chemical indicators of coke wastewater after the treatment processes

Indicator [unit]	Biological treatment	Filtration on sand bed		Reverse osmosis (RO)	
	Value	Value	Retention ratio [%]	Value	Retention coeeficient^*^, *R* [%]
pH	7.2	7.9	–	7.4	–
COD [mg O_2_/L]	6067.4	3692.1	39.1	567.3	84.6
TN [mg NH_4_^+^/L]	334.5	180.2	46.1	9.1	94.9
TOC [mg C/L]	411.1	238.3	42.0	35.2	85.2
TC [mg C/L]	717.5	382.9	46.6	54.1	85.9
SS [mg/L]	132.6	67.0	49.5	1.1	98.4
16PAHs [μg/L]	94.73	45.2	53.3	15.04	67.0

*Retention coefficient was calculated for wastewater taken after filtration on sand bed, and treated in RO process

In many cases, coking wastewater contains high concentration of refractory and toxic compounds and water quality usually cannot meet the discharge standards after conventional biological treatment. According to legal regulations (Journal of law 2014, item. 1800), the wastewater treated in the biological wastewater plant did not meet the quality standards since the concentrations of some selected indicators were too high—COD, TOC.

In this study, a range of pH between 5.2 and 7.9 was selected. The pH of the wastewater after filtration on sand bed was equal to 7.9. During the reverse osmosis, the value of pH decreased to 7.4. In accordane to the polish legalisation, the value of pH did not exceed the permissible values of 6.5–9.0 in the treated wastewater collected to the natural reservoir (Journal of law 2014 item.1800) and the permissible values of 6.5–9.5 in the treated wastewater collected to the severs (Journal of law 2006 no. 136, item. 964). This value of pH is also in the range characteristic for coking wastewater, given by Bartkiewicz (2006).

The value of COD was equal to 6067.4 O_2_/L and decreased to 3692.1 mg O_2_/L after filtration on sand bed and to 567.3 mg O_2_/L after RO. The effectiveness in the removal of the initial COD was 39.1% for filtration on sand bed and 84.6% for reverse osmosis. The final concentration of COD still exceeded the permissible values of treated wastewater. In Zhang et al. (1998) work, removal efficiencies of COD from coking wastewater in anaerobic-anoxic-oxic (A_I_-A_2_-O) fixed biofilm system was equal to 98.8%.

The total nitrogen concentration in wastewater from plant reached 334.5 mg NH_4_+/L. The filtration on sand bed removed approximately 46.1% of TN to value of 180.2 mg NH_4_+/L. The total nitrogen removal efficiency during reverse osmosis was equal to 94.9% (9.1 mg NH_4_+/L).

Following the treatment processes, it showed a decrease of TOC concentration from 411.1 after biological treatment, to 238.3 mg C/L after filtration on sand bed (Removal degree = 42.0%) and to 35.2 mg C/L after RO (Retention coeeficient = 85.2%). TOC removal in RO was similar like in previous study—85.9% (Smol et al. 2014b). The concentration of TC declined after the processes. The effectiveness in TC removal during filtration on sand bed was equal to 46.6% (382.9 mg C/L) and 85.9% after reverse osmosis (54.1 mg C/L).

The concentration of SS reached 132.6 mg/L in bilogically treated wastewater. The 49.5% removal of suspended soils was achieved using after filtration on sand bed (67 mg/L). The concentration of SS declined by 98.4% after RO to 1.1 mg/L.

The initial concentrations of PAHs in the coke wastewater from plant were equal to 94.73 μg/L (Table 5). After the filtration on sand bed, the concentrations of the studied hydrocarbons gradually lowered by 52.3% to the value 45.2 μg/L and after reverse osmosis to value of 5.57 μg/L (Retention coeeficient = 87.7%). In previous studies, the efficiency in 16 EPA PAHs removal from coke wastewater after RO was 89.9% (Smol et al. 2014b) (Table 4).

**Table 4:** Concentration and percentage share of PAHs in coke wastewater treated in biological process, filtration on sand bed and reverse osmosis

PAHs	Biological treatment		Filtration on sand bed		Reverse osmosis (RO)	
	[ng/L]	[%]	[ng/L]	[%]	[ng/L]	[%]
Naf	23,905.59	25.2 ± 4.2	9769.9	21.6 ± 7.1	2911.48	52.3 ± 5.8
Acyl	115.93	0.1 ± 0.1	78.77	0.2 ± 0.1	16.71	0.3 ± 0.1
Ac	7500.43	7.9 ± 2.1	3498.7	7.7 ± 1.0	490.99	8.8 ± 0.9
Fl	302.53	0.3 ± 0.1	245,09	0.5 ± 0.1	76.17	1.4 ± 0.4
Fen	107.99	0.1 ± 0.1	67.22	0.1 ± 0.1	28.21	0.5 ± 0.3
Ant	220.83	0.2 ± 0.1	81	0.2 ± 0.2	9.99	0.2 ± 0.2
Flu	564.34	0.6 ± 0.3	333.91	0.7 ± 0.2	63.45	1.1 ± 0.9
Pir	899.42	0.9 ± 0.2	521.01	1.2 ± 0.3	92.1	1.7 ± 0.1
BaA	13,897.67	14.7 3.2	9032.1	20.0 ± 1.3	762.09	13.7 ± 1.9
Chr	9090.99	9.6 ± 0.8	6721.06	14.9 ± 2.1	859.3	15.4 ± 2.2
BaP	5891.4	6.2 ± 0.5	2981.2	6.6 ± 0.5	67.31	1.2 ± 0.1
BbF	1453.94	1.5 ± 1.0	672.11	1.5 ± 0.3	90.32	1.6 ± 0.9
BkF	24,098.55	25.4 ± 2.4	9091.35	20.1 ± 2.2	96.02	1.7 ± 0.4
DahA	3536.98	3.7 ± 0.4	1018.21	2.3 ± 0.9	2.21	0.0
IP	2190.01	2.3 ± 1.1	645.91	1.4 ± 0.2	0.1	0.0
BghiP	953.47	1.0 ± 0.1	453.07	1.0 ± 0.3	5.32	0.1 ± 0.1
**∑**	94,730.07	–	45,210.61	–	5571.77	–

**Fig. 2:**
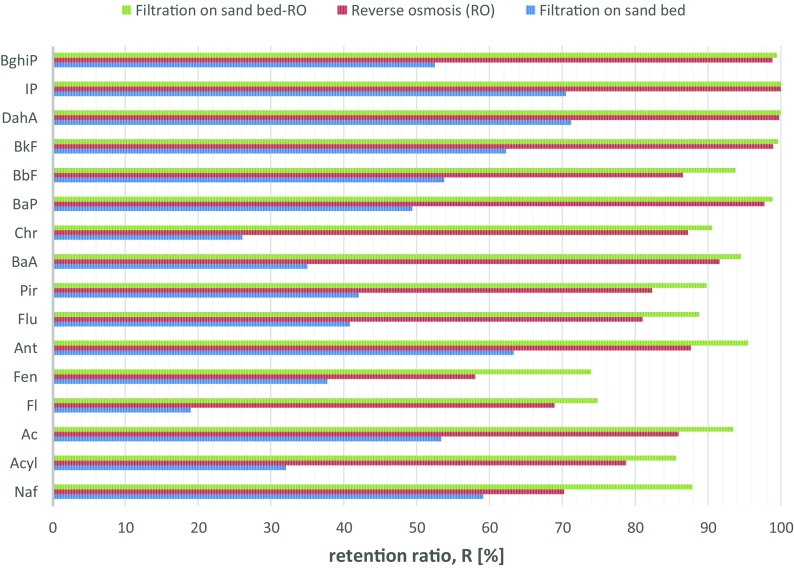
PAHs removal efficiency in treatment processes

The efficiency in removal of individual hydrocarbons was in the 19–100% range (Fig. 2). The average value of the retention coefficient for integrated system filtration-RO was equal to 94.1%. In studies of other authors, the efficiency in removal of PAHs from water in RO reached 88.4% (Bodzek and Konieczny 2011).

The obtained findings indicated that wastewater additionally treated in the process of reverse osmosis still did not meet the standards of quality since the concentration of COD and TOC was high. However, treated wastewater can be converted back and used as technical water in the coke plant, in accordance with a ‘zero waste’ strategy. A zero waste strategy is one of the most visionary concepts (COM 2014, 398) for solving waste problems and assumes that one is moving towards a more circular economy (CE). Transition to a more circular economy requires many changes throughout the value chains, including new ways of turning wastewater into a resource (COM 2015, 614). This implies systemic change, and innovation not only in technologies, but also in the organisation, society, finance methods and policies (Kulczycka and Smol 2016). In industrial networks, zero waste can be understood as a new standard for efficiency and integration (Curran and Williams 2012). A significant improvement in environmental protection is required through the use of the highly effective methods of industrial wastewater treatment that meet the standards and requirements of Polish (Turek et al. 2016) and EU environmental law, defined by the IPPC Directive.

### Phytotoxicity Assessment of Coke Wastewater

To evaluate wastewater toxicity, tests were performed with the test plant *Vicia faba*. The parameters shown in Table 5 (the basic characteristics of the germination inhibition and the degree of toxicity) were used.

**Table 5:** The degree of toxicity (Adamcová et al. 2016)

Inhibition [%]	The degree of toxicity	Evaluation
I* < 10	1	Non-toxic or slightly toxic
10 < *I* < 50	2	Toxic
50 < U	3	Highly toxic

Results obtained from the first experimental block (control sample) show that the germination index, in the case of seeds incubated in cotton matrix was 81.3 ± 3.5, while the germination index for seed incubated in MSBM was 96.7% ± 1.5.

**Fig. 3:**
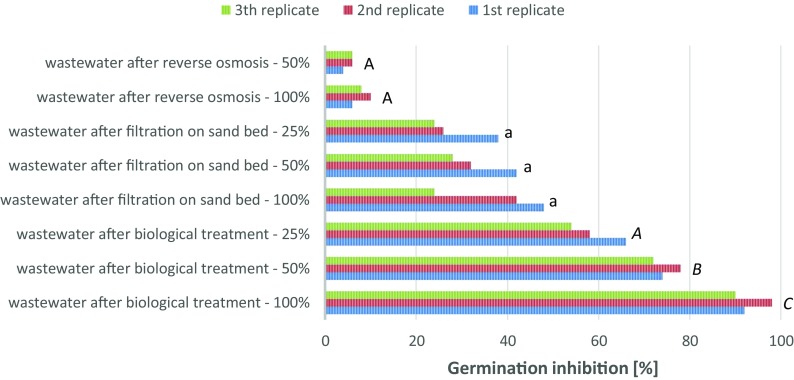
Germination inhibition of *Vicia faba* (cotton matrix)

**Fig. 4:**
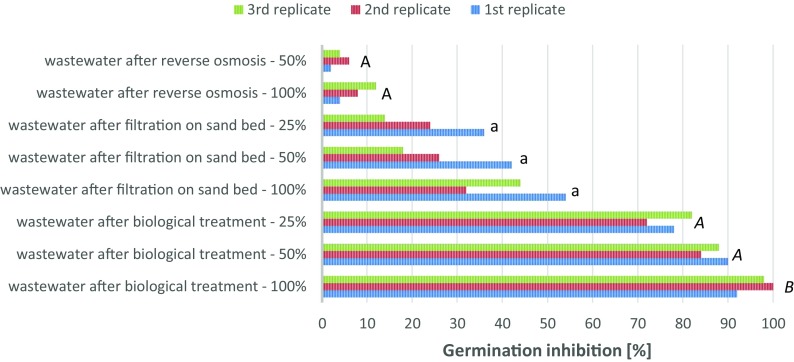
Germination inhibition of *Vicia faba* (Murashige and Skoog Basal Medium matrix)

The phytotoxity of the treated wastewater was evaluated during this investigation. The effects of the coke wastewater (concentration 100%, 50%, 25%) on the inhibition of seed germination of the test plant *Vicia faba* are shown in Figs. 2 and 3 (samples I, II and III). The test results for the cotton matrix are shown in Fig. 3 and for Murashige and Skoog Basal Medium are shown in Fig. 4.

The results of the one-way ANOVA test are presented in Table 6. This analysis was conducted individually on the groups of sampes I, II and III, for both used incubation matrix.

**Table 6:** Results of one-way ANOVA test

	One-way ANOVA *p* value		
	Biological treatment	Filtration on sand bed	Reverse osmosis
Cotton matrix	3.22 10^−4^	0.56	0.12
MSBM matrix	3.38 10^−3^	0.19	0.20

The inhibition of germination (%) of *Vicia faba* in sample I—wastewater (concentration 100%) collected from the biological treatment plant was in the range of 90–98% for a cotton matrix and 92–100% for the Murashige and Skoog Basal Medium matrix. These results confirmed that coke wastewater was insufficiently treated following the biological process and was strongly toxic to the test plant—the degree of toxicity was 3. The germination inhibition for the diluted wastewater (concentration 50%) was in the range of 72–78% for the cotton matrix and 84–90% for the MSBM matrix. These samples are strongly toxic, the degree of toxicity is 3. For a concentration of 25%, the germination inhibition was still in the range for strongly toxic wastewater (degree of toxicity 3) and was equal to 66% for the cotton matrix and 82% for the MSBM matrix.

The germination inhibition of the test plants in sample II—wastewater filtered on a sand bed (concentration 100%) was equal to 48% for the cotton matrix and 54% for the commercial MSBM matrix. The germination inhibition decreased on dilution of the sample. The germination inhibition for the diluted wastewater (concentration 50%) was equal to 42% for the cotton and MSBM matrices. The germination inhibition for the 25% concentration of wastewater was in the range of 24–38% for cotton and 14–36% for the MSBM matrix. The results confirmed that wastewater, even after treatment on a sand bed, was still toxic with a degree of toxicity of 2.

In the last phase of the research, wastewater treated in reverse osmosis was examined. The germination inhibition of *Vicia faba* for sample III (concentration 100%) was in the range of 6–10% for cotton and 4–12% for the MSBM matrix. Sample III was diluted to 50% concentration. This contributed to a decrease in the germination inhibition value. The germination inhibition was in the range of 4–6% for the cotton matrix and 2–6% for the MSBM matrix. These samples are non-toxic or slightly toxic for the test plant, the degree of toxicity is 1.

The results presented in Table 4 indicate that all analysed samples show a statistically valid differences between the level of germination inhibition, within groups. The large differences can be observed in samples with the addition of wastewater treated in the biological processes. This result may mean that phytotoxicity of wastewater not treated by filtration techniques is highly dependent on the dose of this waste. This conclusion is also confirmed by post hoc Tukey’s test. This additional analysis, of individual differences between single means, within groups of samples, shows that only in case of samples with the addition of wastewater after biological treatment the statistical valid differences can be observed.

Based on the tests conducted, significant, linearly increasing toxicity of coke wastewater was indicated. In all series, correlation is observed between an increasing concentration of wastewater and the inhibition of growth of the test plant. Such a relationship has also been demonstrated by other authors. In the work of Adamcová et al. (2016), the plant species of the Phytotoxkit microbiotest responded differently to the degree of contamination of the sewage sludge samples with a concentration 100, 50, 25 and 10%. Growth inhibition values clearly revealed the inhibitory effects of sewage sludge contaminants on seed germination and root elongation of *Sinapis alba* L. (Adamcová et al. 2016).

The present study highlights the need to monitor not only the basic physical and chemical indicators (including the level of toxic substances such as PAHs), but also their effect on test organisms. Despite the fact that wastewater was treated in a biological installation, it still contains substances strongly hazardous to health and life—the degree of toxicity reached 3. After filtration on a sand bed, the reduction of selected indicators was equal to 39% for COD, 46% for TN, 42% for TOC, 47% for TC, 50% for SS and 53% for toxic PAHs. However, wastewater still showed a toxic effect—the degree of toxicity reached 2. Reverse osmomis was the most effective process in the removal of the indicators analysed. The concentration of COD decreased by 85%, TN—95%, TOC—85%, TC—85%, SS—98% and sum of 16PAHs—67%.

The effect of wastewater and sludge on seed germination and plant growth has been the subject of research of numerous researchers (Adamcová et al. 2016). The assessment of the genotoxicity of coke wastewater was studied by Sindera et al. (2011). The authors indicated that despite the fact that a reduction of TOC and COD of about 90% was noted in the treated coke wastewater, it still contains substances toxic to health and life. The paper presents the results of tests of raw and biologically treated wastewater for its phyto- and genotoxicity for *Vicia faba*. It was observed that with an increasing concentration of wastewater, germination inhibition also increases. The germination inhibition was in the range of 65–79% for a 30% concentration of treated wastewater and 70–80% for a 60% concentration. Raw coke wastewater showed a significantly greater effect on the test plant as compared with the samples obtained from the effluent. The growth inhibition was equal to 89% (Sindera et al. 2011). In the research of Khoufi et al. (2006), an integrated technology for the treatment of the recalcitrant contaminants of olive mill wastewater was examined. The method involves an electrochemical step for the pre-treatment of wastewater using the electro-Fenton reaction followed by an anaerobic bio-treatment. The authors indicated that the electro-Fenton process removed 65.8% of the total polyphenolic compounds and subsequently decreased the wastewater toxicity from 100 to 66.9%, which resulted in improving the performance of the anaerobic digestion. In the combined process, a high overall reduction in COD, suspended solids, polyphenols and lipid content was achieved by the two successive stages. The authors emphasise that the result opens promising perspectives since its use in the process concept as a fast and cheap pre-treatment prior to conventional anaerobic post-treatment through electro-coagulation as post-treatment technology completely detoxified the anaerobic effluent and removed its toxic compounds (Khoufi et al. 2006). The genotoxicity of coking wastewater was studied using *Vicia faba* and *Hordeum vulgare* root tip cytogenetic bioassays by Dong and Zhang (2010). Results showed that the coke wastewater decreased the mitotic index, and significantly enhanced the frequencies of micronucleus, sister chromatid exchange and pycnotic cell in concentration-dependent manners. Exposure to the same wastewater concentration, the increasing ratios of above genetic injuries were higher in *Vicia faba* than that in *Hordeum vulgare* (Dong and Zhang 2010). *Vicia faba* is more sensitive for toxic environment and should be used in toxity tests. The toxicity of coke wastewater treated with advanced oxidation by the Fenton process supported by an ultrasonic field was studied by Kwarciak-Kozłowska and Krzywicka (2016). Two doses of iron (4 g/L and 40 g/L) and four doses of hydrogen peroxide (an amount proportional to the value of the COD of raw wastewater, ranging from a COD/H_2_O_2_ ratio of 1:2.5 to 1:20) were used. Two tests, an algal growth inhibition test and a *Lepidium sativum* test, were used to determine the toxicity of coke wastewater. The authors indicated that higher COD and TOC value reductions were obtained after application of a higher dose of ferrous sulphate (40 g/L). Higher toxicity to *Lepidium sativum* was observed when a higher dose of ferrous sulphate and the lowest dose of hydrogen peroxide were introduced to wastewater samples. On the other side, higher toxicity to algae was observed for a lower dose of ferrous sulphate. In this case, increasing the dose of hydrogen peroxide resulted in a decrease in toxicity (Kwarciak-Kozłowska and Krzywicka 2016). Phytotoxicity tests are also used in order to check the effectivness of phytoremediation as an in situ method. Al-Baldawi et al. (2013) conducted phytotoxicity tests on *Scirpus grossus* on contaminated water at different diesel concentrations (0, 8700, 17,400 and 26,100 mg/L). The percentage degradation of total petroleum hydrocarbons (TPH) by the test plant was recorded from the extraction of synthetic wastewater with plants and the corresponding control contaminant without plants during the 72-day treatment period. After this period of wastewater treatment in a subsurface flow system, authors indicated that *S. grossus* has the capability to survive and provide good conditions for rhizobacteria to degrade hydrocarbon at all the diesel concentrations investigated (Al-Baldawi et al. 2013). In the current paper, evaluation of toxicity of coke wastewater was carried out for the plant organisms, but the analysis can also be expanded taking into account other organisms, e.g. fish and animals. Zhou et al. (2015) conducted a battery of toxicity tests using photo bacterium, algae, crustacean and fish to evaluate acute toxicity profile of coking wastewater after the novel wastewater treatment process—vertical tubular biological reactor (VTBR). Authors indicated decrease in toxicity of coking wastewater after VTBR: Toxicity Unit (TU) decreased from 21.2 to 0.4 for *Photobacterium phosphoreum*, from 9.5 to 0.6 for *Isochrysis galbana*, from 31.9 to 1.3 for *Daphnia magna*, and from 30.0 to nearly 0 for *Danio rerio*. A battery of toxicity tests are mentioned as useful tool for the clarity of toxicity profile for complex environmental samples (including coke wastewater) using different aquatic test organisms as photo bacterium, algae, crustacean and fish (Zhou et al. 2015). Further study should be conducted in this area.

## Conclusions

The purpose of the research was to monitor the effectivnes in the removal of selected pollutants from coke wastewater in the integrated membrane processes and to determine the impact of coke wastewater on the germination of the test plant, broad bean (*Vicia faba*), on two matrices: cotton and Murashige and Skoog Basal Medium—MSBM. The results confirm that coke wastewater is effectively treated in presented system: filtration on sand bed—reverse osmosis, and there is a noticeable correlation between increasing concentrations of wastewater and the seed germination of the test plant.

During the investigation, a high removal efficiency was obtained for the selected indicators: after filtration through a sand bed the reduction of COD was 39%, TN—46%, TOC—42%, TC—47%, SS—50%, 16PAHs—53% and after RO: reduction of COD—85%, TN—95%, TOC—85%, TC—85%, SS—98%, 16PAHs—67%. However, there is a necessity to not only monitor the basic physical and chemical indicators, but also their effect on the test organisms.

Coke wastewater collected from the biological treatment plant showed very high levels of germination inhibition (90–98% for the cotton matrix and 92–100% for the MSBM matrix). These samples are indicated as strongly toxic with a degree of toxicity of 3.

Wastewater, even after treatment on a sand bed, was still toxic. Its germination inhibition was in the range of 24–48% for a cotton matrix and 14–54% for an MSBM matrix, and the degree of toxicity was equal to 2.

The toxicity of wastewater was lowest when it was treated in a reverse osmosis process. The germination inhibition was in the range of 4–10% for a cotton matrix and 2–12% for an MSBM matrix. Wastewater after RO is non-toxic or slightly toxic to the test plant, the degree of toxicity does not exceed 1.

Biologically treated wastewater showed significantly greater phytotoxic effects compared with those obtained in the effluent treated on a sand bed and in reverse osmosis.
